# Cost of in-patient management of COVID-19 patients in a general hospital in Kuwait

**DOI:** 10.1186/s12913-023-10287-z

**Published:** 2023-11-28

**Authors:** Amrizal Muhammad Nur, Syed Mohamed Aljunid, Mohammad Almari

**Affiliations:** 1 Department of Health Policy and Management, College of Public Health, Kuwait University, Shadadiya, Kuwait; 2 Department of Public Health and Community Medicine, International Medical University, Kuala Lumpur, Malaysia

**Keywords:** Cost, COVID-19, Hospital

## Abstract

**Background:**

Among the GCC countries affected by COVID-19 infections, Kuwait has been significantly impacted, with 658,520 cases and 2,563 deaths reported by the WHO on September 30, 2022. However, the impact of the COVID-19 epidemic on Kuwait’s economy, especially in the healthcare sector, remains unknown. Objective: This study aims to determine the total cost of managing COVID-19 in-patients in Kuwait.

**Method:**

A cross-sectional design was employed for this study. A total of 485 COVID-19 patients admitted to a general hospital responsible for COVID-19 cases management were randomly selected for this study from May 1st to September 31st, 2021. Data on sociodemographic information, length of stay (LOS), discharge status, and comorbidities were obtained from the patients’ medical records. The data on costs in this study cover administration, utility, pharmacy, radiology, laboratory, nursing, and ICU costs. The unit cost per admission was calculated using a step-down costing method with three levels of cost centers. The unit cost was then multiplied by the individual patient’s length of stay to determine the cost of care per patient per admission.

**Findings:**

The mean cost of COVID-19 in-patient care per admission was KD 2,216 (SD = 2,018), which is equivalent to USD 7,344 (SD = 6,688), with an average length of stay of 9.4 (SD = 8.5) days per admission. The total treatment costs for COVID-19 in-patients (n = 485) were estimated to be KD 1,074,644 (USD 3,561,585), with physician and nursing care costs constituting the largest share at 42.1%, amounting to KD 452,154 (USD 1,498,529). The second and third-largest costs were intensive care (20.6%) at KD 221,439 (USD 733,893) and laboratory costs (10.2%) at KD 109,264 (USD 362,123). The average cost for severe COVID-19 patients was KD 4,626 (USD 15,332), which is almost three times higher than non-severe patients of KD 1,544 (USD 5,117).

**Conclusion:**

Managing COVID-19 cases comes with substantial costs. This cost information can assist hospital managers and policymakers in designing more efficient interventions, especially for managing high-risk groups.

**Supplementary Information:**

The online version contains supplementary material available at 10.1186/s12913-023-10287-z.

## Introduction

Initial cases of novel coronavirus (2019-nCoV)-infected pneumonia (NCIP) were reported in Wuhan, Hubei Province, China, in December 2019 and January 2020 [1]. This virus has resulted in widespread pandemics with high morbidity and mortality [2]. Globally, the World Health Organization (WHO) reported 614,385,693 confirmed cases of COVID-19, including 6,522,600 deaths and 325,602 new cases on September 27, 2022. Additionally, a total of 12,677,499,928 vaccine doses were administered [3]. Among the Gulf Cooperation Council (GCC) countries affected by COVID-19 infections, Kuwait has been impacted, with 658,520 cases and 2,563 deaths. As of September 30, 2022, 8,214,656 vaccine doses have been administered in Kuwait [4].

Many countries closed their borders, suspended flights, and reduced international interactions. These measures caused a significant worldwide economic downturn, leading to business closures, increased unemployment, rising inflation, interrupted production, and shipping [5]. Quarantines, regional lockdowns, and social distancing measures were adopted to contain the virus. The impact of COVID-19 on the global economy has been severe, as these infections led to reduced labor supply, workplace closures, decreased productivity, layoffs, income declines, fear of contagion, heightened uncertainty, and further business closures and job losses. All these factors contributed to a substantial portion of the economy shutting down, as acknowledged by the International Monetary Fund (IMF) [6]. World leaders had already received warnings about the sustainability of healthcare systems before the current pandemic [7, 8]. The rapid and global spread of the pandemic exacerbated existing problems and created new challenges for decision-makers, negatively affecting public health [9]. To mitigate the impact of this and future pandemics, decision-makers must understand how their health systems are affected. Of particular importance is understanding healthcare resource utilization, such as the length of hospital stays, and the subsequent costs of the pandemic [10].

Kuwait, a Middle Eastern country with a population of 4,328,553 million (2021), has a GDP per capita estimated at USD 24,811, and the current health expenditure is 5.5% of the GDP [11, 12]. Estimating the burden of coronavirus disease on the health system can assist policymakers in effectively allocating resources, prioritizing measures, and emphasizing the importance of long-term planning for sustainable financing in similar conditions. To the best of our knowledge, the impact of the COVID-19 epidemic on Kuwait’s economy, particularly in the health sector, remains unknown. This is the first study on health economic evaluation in Kuwait, which aims to determine the total cost of COVID-19 in-patient management in a general government hospital.

## Methods

This study employed a cross-sectional design to assess the management cost of COVID-19 in-patients with positive PCR test results in a general hospital in Kuwait from May 1st to September 31st, 2021. Clinical data from 485 randomly selected medical records (MR) were obtained and extracted from electronic patient records through the Hospital Information System (HIS) using a patient data collection tool (see appendix 1). The estimated minimum sample size was based on the total number of cases (14,518) with positive COVID-19 admitted in the general hospital on May 1, 2021, as reported by medical record department. The sample size was determined using Slovin’s formula (Galero-Tejero,2011). The Slovin’s formula assumes a degree of variability (proportion) of 0.05 and a confidence level of 95%. All patient admitted to the general hospital with confirmed COVID-19 diagnosis and discharged from 1st February to 30th April 2021 was used to select cases to be included in this study. The patient clinical data included sociodemographic characteristics (age, sex, etc.), length of stay (LOS), discharge status, primary diagnosis, comorbidity, medical or surgical procedures, laboratory tests, radiology tests, and physiotherapy services. The management cost of COVID-19 patients was computed using a step-down costing method from the provider’s perspective. Hospital costing data were collected using the costing data collection tool in an excel sheet format that organized costing data into three levels of cost centers: overhead cost centers (e.g., administration, consumables, maintenance, etc.), intermediate cost centers (e.g., pharmacy, radiology, etc.), and final cost centers, including all wards and clinics (see appendix 2). To estimate the cost for each cost center, both capital costs (building, equipment, and furniture costs) and recurrent costs (staff salary and other operational costs) were combined. Information on activities that reflect the workload, such as the number of discharges, inpatient days, number of visits, and floor space, were gathered from medical records and engineering departments to determine appropriate allocation factors.

### Computing of the provider cost

The computation of the provider cost involved seven steps in the step-down costing approach:

1. Defining the cost centers.
2. Grouping cost centers into overhead cost centers, intermediate cost centers, and final care cost centers.
3. Obtaining the total cost of each overhead, intermediate, and final care cost center.
4. Deciding on units to distribute costs.
5. Allocating the costs of overhead cost centers to intermediate and final care cost centers.
6. Allocating the costs of intermediate-to-final care cost centers.
7. Relating the unit cost to the individual patient’s length of stay to obtain the cost of care per patient per admission.

These seven steps were adopted and modified from WHO, Shepard, DS et al. (2000) [13] and have been used for similar studies by Amrizal et al. [14, 15].

## Results

### Patient demographics and other characteristics

A total of 485 hospitalized patients with COVID-19 were included in this study. The mean age and standard deviation (SD) of patients were estimated to be 52.9 (SD = 18.1) years, with a median age of 55 years. Most of the patients were Kuwaiti (87.0%), and the majority were females (54.6%). They were either admitted to the general ward (79.6%) or the ICU (20.4%) and were discharged home or recovered (73.4%). The majority of the patients were symptomatic (94.8%). The mean length of hospital stay was 9.4 ± 8.5 days. COVID-19 disease types were categorized as mild (28.5%), moderate (53.1%), and severe (18.4%) [see table-1]. The doctor in-charge of treating the COVID-19 patient assigned the severity level of the case based on the criteria set by the WHO [3] as follows:

**Table 1:** Demographics and other characteristics of COVID-19 Patients

Patient Demographic and Characteristics	Mild (N = 139)	Moderate (N = 259)	Severe (N = 87)	Overall (N = 485)
**Gender**				
Female	78 (56.1%)	146 (56.4%)	41 (47.1%)	265 (54.6%)
Male	61 (43.9%)	113 (43.6%)	46 (52.9%)	220 (45.4%)
**Age (years)**				
Mean ± SD	47.2 ± 19.1	53.7 ± 17.6	59.9 ± 14.9	52.9 ± 18.1
Median [Min-Max]	50 (0–89)	55 (0–97)	60 [15–94]	55 [0–97]
**LOS (days)**				
Mean ± SD	4.5 ± 3.4	8.6 ± 5.2	19.6 ± 12.8	9.4 ± 8.5
Median [Min-Max]	4 [1–29]	7 [1–43]	17 [2–57]	7 [1–57]
**Nationality**				
Kuwaiti	130 (93.5%)	229 (88.4%)	63 (72.4%)	422 (87.0%)
Non-Kuwaiti	9 (6.5%)	30 (11.6%)	24 (27.6%)	63 (13.0%)
**Admission**				
General ward	138 (99.3%)	245 (94.6%)	3 (3.4%)	386 (79.6%)
ICU	1 (0.7%)	14 (5.4%)	84 (96.6%)	99 (20.4%)
**Disease Type**				
Asymptomatic	8 (5.8%)	7 (2.7%)	10 (11.5%)	25 (5.2%)
Symptomatic	131 (94.2%)	252 (97.3%)	77 (88.5%)	460 (94.8%)
**Disease Severity**	139 (28.5%)	259 (53.1%)	87 (18.4%)	485 (100%)
**Discharge Status**				
Recovered	121 (87.1%)	223 (86.1%)	12 (13.8%)	356 (73.4%)
Transfer to other hospital	1 (0.7%)	9 (3.5%)	21 (24.1%)	31 (6.4%)
DAMA	17 (12.2%)	25 (9.7%)	0 (0.0%)	42 (8.7%)
Died	0 (0.0%)	2 (0.8%)	54 (62.1%)	56 (11.5%)
Total	139 (28.7%)	259 (53.4%)	87 (17.9%)	485 (100%)

Notes: - ICU= Intensive Care Unit, LOS = Length of Stay and SD = Standard Deviation

- Mild disease: Symptomatic COVID-19 patients without evidence of viral pneumonia or hypoxia.
- Moderate disease: Adolescents or adults with clinical signs of pneumonia (fever, cough, dyspnea, fast breathing) but no signs of severe pneumonia, including SpO2 ≥ 90% on room air.
- Severe disease: Adolescents or adults with clinical signs of pneumonia (fever, cough, dyspnea) plus one of the following: respiratory rate > 30 breaths/min, severe respiratory distress, or SpO2 < 90% on room air.

### Provider cost

As shown in Table 2, the treatment cost per admission and average length of stay (ALOS) for severe conditions were higher than those for moderate and mild conditions. The treatment cost per admission and ALOS for severe conditions were KD 4,626 (SD = 3,035), equivalent to USD 15,331 (SD = 10,059), and 19.6 days (SD = 12.8), respectively. Current US dollars were used for international comparison, with an average exchange rate in 2021 (1 KD = USD 3.3142).

**Table 2:** Mean Cost and Length of Stay (LOS) of COVID-19 Patients by Severity

Types of Severity	N	Mean LOS (SD) (days)	Mean Cost (KD)	SD (KD)	Mean Cost (USD)	SD (USD)
Mild	139	4.5 (SD = 3.4)	1,064	795	3,526	2,635
Moderate	259	8.6 (SD = 5.2)	2,024	1,233	6,708	4,086
Severe	87	19.6 (SD = 12.8)	4,626	3,035	15,331	10,059
Averages	485	9.4 (SD = 8.5)	2,216	2,018	7,344	6,688

Notes: SD = Standard Deviation. KD = Kuwaiti Dinar. USD = US Dollar. Average exchange rate in year 2021 (1 KD = 3.3142 USD). LOS = Length of Stay. N = Number of patients

As shown in Table 3, the treatment cost per admission and ALOS for patients in the ICU were higher than those for patients in the general ward. The treatment cost per admission and ALOS for ICU patients were as follows: KD 4,342 (SD = 2,992), equal to USD 14,390 (SD = 9,916), and 18.4 days (SD = 12.7), respectively.

**Table 3:** Mean Cost and Length of Stay (LOS) of COVID-19 Patients by General Ward and ICU

Types of Admission	N	Mean LOS (SD) (days)	Mean Cost (KD)	SD (KD)	Mean Cost (USD)	SD (USD)
General Ward	386	7.1 (SD = 4.9)	1,670	1,175	5,535	3,894
ICU	99	18.4 (SD = 12.7)	4,342	2,992	14,390	9,916
Averages	485	9.4 (SD = 8.5)	2,216	2,018	7,344	6,688

Notes: SD = Standard Deviation. KD = Kuwaiti Dinar. USD = US Dollar. Average exchange rate in year 2021 (1 KD = 3.3142 USD). LOS = Length of Stay. N = Number of patients

**Fig. 1:**
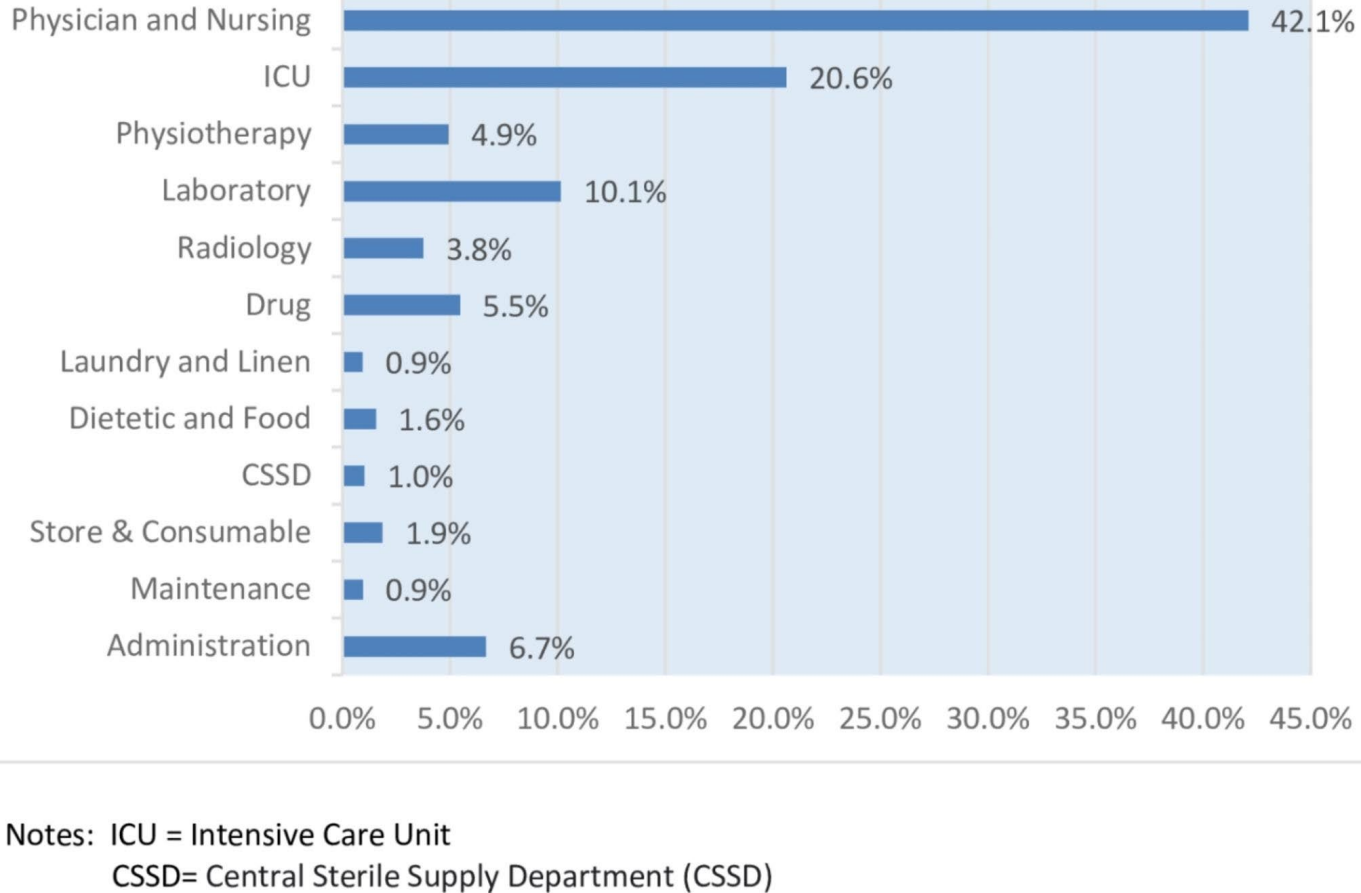
The Percentage of Cost Component in Managing COVID-19 Patients

Regarding the total treatment costs and the percentages of cost components for the 485 COVID-19 in-patients, they were estimated to be KD 1,074,644 (USD 3,561,585). The physician and nursing care costs constituted the largest share of the costs (42.1%), amounting to KD 452,154, equivalent to USD 1,498,529. Following that, the primary costs were intensive care costs (20.6%), totaling KD 221,439, equal to USD 733,893, and laboratory costs (10.2%), totaling KD 109,264, equivalent to USD 362,123. The average treatment cost for COVID-19 patients with severe conditions was nearly three times higher than that for non-severe patients. The average cost for severe patients was KD 4,626 (SD = 3,035), equivalent to USD 15,332 (SD = 10,059), while non-severe patients had an average cost of KD 1,544 (SD = 1,014), equal to USD 5,117 (SD = 3,361) (Table 4; Fig. 1).

**Table 4:** Total Cost of Each Cost Component by Severe and Non-Severe of COVID-19 Patients

Cost Component	%	Total Cost All Patient (n = 485) (KD/USD)	Mean Cost per Patient (KD/USD) ±(SD)	SD	Mean Cost per Non-Severe Patient (KD/USD) ±(SD)		SD	Mean Cost per Severe Patient (KD/USD) ± (SD)		SD
Administration	6.7%	71,479 (KD)	147 (KD)	134 (KD)		103 (KD)	68 (KD)		308 (KD)	202 (KD)
		236,896 (USD)	487 (USD)	444 (USD)		341 (USD)	225 (USD)		1,021 (USD)	669 (USD)
Maintenance	0.9%	10,094 (KD)	21 (KD)	19 (KD)		15 (KD)	10 (KD)		43 (KD)	28 (KD)
		33,454 (USD)	70 (USD)	63 (USD)		50 (USD)	33 (USD)		143 (USD)	93 (USD)
Store & Consumable	1.9%	20,325 (KD)	42 (KD)	38 (KD)		29 (KD)	19 (KD)		87 (KD)	57 (KD)
		67,361 (USD)	139 (USD)	126(USD)		96 (USD)	63 (USD)		288 (USD)	189 (USD)
CSSD	1.0%	11,186 (KD)	23 (KD)	21 (KD)		16 (KD)	11 (KD)		48 (KD)	32 (KD)
		37,073 (USD)	76 (USD)	70 (USD)		53 (USD)	36 (USD)		159 (USD)	106 (USD)
Dietetic and Food	1.6%	17,057 (KD)	35 (KD)	32 (KD)		25 (KD)	17 (KD)		73 (KD)	48 (KD)
		56,530 (USD)	116 (USD)	106 (USD)		83 (USD)	56 (USD)		242 (USD)	159 (USD)
Laundry and Linen	0.9%	9,958 (KD)	20 (KD)	19 (KD)		15 (KD)	9 (KD)		43 (KD)	28 (KD)
		33,003 (USD)	66 (USD)	63 (USD)		50 (USD)	30 (USD)		143 (USD)	76 (USD)
Drug	5.5%	58,611 (KD)	121 (KD)	110 (KD)		84 (KD)	55 (KD)		252 (KD)	165 (KD)
		194,249 (USD)	401 (USD)	365 (USD)		278 (USD)	182 (USD)		835 (USD)	547 (USD)
Radiology	3.7%	40,286 (KD)	83 (KD)	76 (KD)		58 (KD)	38 (KD)		173 (KD)	114 (KD)
		133,516 (USD)	275 (USD)	252 (USD)		192 (USD)	126 (USD)		573 (USD)	378 (USD)
Laboratory (III)	10.2%	109,264 (KD)	225 (KD)	205 (KD)		157 (KD)	103 (KD)		470 (KD)	309 (KD)
		362,123 (USD)	746 (USD)	679 (USD)		520 (USD)	341 (USD)		1558 (USD)	1,024 (USD)
Physiotherapy	4.9%	52,791 (KD)	109 (KD)	99 (KD)		76 (KD)	50 (KD)		227 (KD)	149 (KD)
		174,960 (USD)	348 (USD)	328 (USD)		252 (USD)	166 (USD)		752 (USD)	494 (USD)
ICU (II)	20.6%	221,439 (KD)	457 (KD)	416 (KD)		318 (KD)	209 (KD)		953 (KD)	625 (KD)
		733,893 (USD)	1515 (USD)	1379 (USD)		1,054 (USD)	693 (USD)		3,158 (USD)	2,071 (USD)
Physician and Nursing (I)	42.1%	452,154 KD	932 (KD)	849 (KD)		650 (KD)	427 (KD)		1,946 (KD)	1,277 (KD)
		1,498,529 (USD)	3,089 (USD)	2,814 (USD)		2,154 (USD)	1,415 (USD)		6,449 (USD)	4,232 (USD)
Average Cost	100%	1,074,644 (KD)	2,216 (KD)	2,018 (KD)		1,544 (KD)	1,014 (KD)		4,626 (KD)	3,035 (KD)
	3,561,585 (USD)		7,344 (USD)	6,688 (USD)		5,117 (USD)	3,361 (USD)		15,332 (USD)	10,059 (USD)

Notes: SD = Standard Deviation. KD = Kuwaiti Dinar. USD = US Dollar. Average exchange rate in year 2021 (1 KD = 3.3142 USD). LOS = Length of Stay. N = Number of patient

## Discussion

The outbreak of COVID-19 and the increasing number of patients in Kuwait impose high costs on infected patients, their families, and the healthcare system. The economic burden of these diseases has been a cause of concern among managers and policymakers in the health sector. Therefore, identifying the economic consequences of COVID-19 could provide valuable evidence for policymaking. This study provides an estimate of the cost of COVID-19 per patient per admission from a healthcare provider’s perspective. We report an average inpatient treatment cost of USD 7,344, which is almost similar to the cost in China of USD 6,827 from 70 empirically observed cases [16], but lower than the cost reported in Saudi Arabia (USD 12,547) [17], and lower than that reported in a recent study conducted by Shrestha et al. (2021), where the average hospitalization cost per patient was estimated to be USD 13,090 [18]. This current finding is higher compared to several other studies conducted by Bartsch et al. in the USA [19], where the cost of COVID-19 per admission ranged from USD 2,837 to USD 3,205, Gedik et al. in Turkey [20], where the mean cost of COVID-19 patients was USD 882 (SD = 667), and Ghaffari Darab et al. in Iran [21], where the mean cost of COVID-19 patients was USD 3,755. Meanwhile, the mean cost of mild, moderate, and severe COVID-19 in this study were USD 3,526, USD 6,708, and USD 15,331, respectively. These findings are lower compared to the study conducted by Li et al. in China; the mean cost of mild COVID-19 and moderate COVID-19 were USD 4,552 and USD 11,058, respectively [16]. In this study, it was found that more severe COVID-19 patients and ICU patients had higher healthcare costs than milder COVID-19 patients. This finding is similar to studies by Athanasakis et al. [22], Gedik et al. [20], Li et al. [16], and Khan et al. [17]. Regarding the cost comparison between the general ward and ICU admission, the treatment cost per admission in the general ward and ICU admission was USD 5,535 and USD 14,390, respectively. This finding is lower than the study in Saudi Arabia, where the cost of cases managed in the general Medical Ward (GMW) and ICU were USD 11,385 and USD 21,173, respectively [23]. This finding is also lower than that of a recent study conducted in the United States by Shrestha et al. (2021), where the average hospitalization costs per patient were estimated to be USD 13,090 without ICU and USD 21,222 with ICU admission [18]. The cost comparisons mentioned earlier might be influenced by the countries’ health system, costing methodologies and criteria in selection of cases in the studies. However, through the cost comparisons, we can appreciate the trend and differences in the various cost components of managing COVID-19 cases. Additionally, we can identify the major cost components in managing COVID-19 patients and plan for more efficient management of these high-cost items. Thus, this cost information can assist the top-level management of the hospital in planning for more efficient and effective resource allocation in managing cases of COVID-19 especially during the pandemic. There have been significant differences in the cost of healthcare delivery units worldwide in non-coronavirus circumstances [24]. However, such comparisons can provide an understanding of the severity of financial effects on the health systems of other countries. As shown in Table 3, the average cost of patients staying in the ICU (USD 14,390) is about 3 times that of patients who did not receive intensive care (USD 5,535). In the study population of the present study, 20.4% (99/485) of hospitalized patients used intensive care, while in the study by Darab et al., this rate was 7% [21]. Some other studies have suggested that about one-third of people infected with SARS-CoV-2 are in critical condition and require intensive care [25]. Others report that approximately 5% of COVID-19-confirmed cases require intensive care [26].

Based on the results presented in Table 4, the total treatment costs of COVID-19 inpatients were estimated to be KD 1,074,644, equivalent to USD 3,561,585, in which physician and nursing care costs accounted for the largest share of costs (42.1%). The other costs included intensive care costs (20.6%; USD 733,893), laboratory costs (10.2%; USD 362,123), administration costs (6.7%; USD 236,896), and drug costs (5.5%; USD 194,249). This highest cost component is different from the Ghaffari Darab study, where the largest share of costs was associated with intensive care and nursing services at 43% of total costs [21]. However, in the study by Li X-Z, the highest costs were related to medicines [16]. In this study, the average Length of Stay (ALOS) per patient per admission averaged 9.4 days (SD = 8.5). This ALOS is lower than a study conducted in Germany, which showed that the average length of stay in the hospital for all patients was 14.3 days [27]. The estimates in the present study indicate that the average cost in the group with severe conditions was approximately three times that of the group with mild conditions.

The mean ± SD age of patients was estimated at 52.9 ± 18.1 years, with a median age of 55 years. Similar to Richardson’s study, it was also confirmed that among hospitalized patients with COVID-19, most were elderly men [28]. In a study conducted in China, hospitalized patients were mostly men, with a median age of 56 years, and 26% required inpatient care [29]. As previously mentioned, the COVID-19 outbreak has already imposed massive costs on the Kuwait healthcare system, and the disease response system must adapt to the financial shock caused by this disease in various ways. Ensuring a comprehensive response to the COVID-19 pandemic requires public funding. Reprioritizing public expenditure to strengthen the health and economic system requires timely measures by government leaders and a supportive public funding environment. To respond to these new economic and financial constraints, adjustments must be made to both the revenue and expenditure dimensions of the budget [29]. In this study, we only investigated the costs of hospitalized patients, excluding other cost dimensions in the health system and the economy of the country. Given the unpredictable nature of this disease and its definitive treatment, the development of evidence in the field of epidemiological dimensions and its economic effects requires appropriate decision-making and policymaking, and research efforts in this field should be continued.

### Study limitation and recommendation

This study was conducted in a single general hospital designated by the government as a center for COVID-19 patient management. The cost was calculated from the hospital’s perspective, excluding indirect costs such as lost income due to hospitalization, lost income due to recovery at home, and potential productivity loss due to premature death. The cost comparison was not straightforward due to difficulties in comparing the literature across various methodologies, populations, healthcare costs, health systems, and more. Nevertheless, the study findings were analyzed using established, standardized, and reproducible methods with the aim of supporting emergency preparedness in future referral hospitals. However, these findings did not encompass community-based care costs, PPE equipment costs, transport costs, surveillance efforts, or other impacts on the healthcare system. It is recommended that further studies examine and explore a comprehensive cost analysis, encompassing indirect costs such as lost income during hospitalization, lost income during recovery at home, and potential productivity loss due to premature death. Additionally, other costs such as PPE equipment costs, transport costs, out-of-pocket expenses for patients, home quarantine costs, institutional quarantine costs, and vaccination costs should be included. It is also suggested that the government should invest in prevention strategies rather than treatment approaches to control these diseases.

## Conclusion

The average treatment cost per patient per admission was KD 2,216 (SD = 2,018), equivalent to USD 7,344 (SD = USD 6,688). The average Length of Stay (ALOS) per patient per admission was 9.4 days (SD = 8.5). Physician and nursing care costs accounted the largest share of costs (42.1%; USD 1,498,529), followed by Intensive care costs (20.6%; USD 733,893) and laboratory costs (10.2%; USD 362,123). The average cost for patients with severe conditions was approximately three times that of patients with mild conditions. This cost information can assist hospital managers and policymakers in designing more efficient interventions, particularly for the management of high-risk groups.

### Electronic supplementary material

Below is the link to the electronic supplementary material.

**Appendix-1**: Patient Data Collection Form

**Appendix-2**: Costing Data Collection Tool

## Data Availability

The data that support the findings of this study are available from a selected General Hospital, but restrictions apply to the availability of these data and are not publicly available. However, data are available from the corresponding author upon reasonable request and with permission from a selected general Hospital.
