# Supplementary material for: Cost of in-patient management of COVID-19 patients in a general hospital in Kuwait
Source: BMC Health Serv Res. 2023 Nov 28;23:1314. doi: 10.1186/s12913-023-10287-z (PMC10685622; doi:10.1186/s12913-023-10287-z)
Supplement: Supplementary file 1 — Appendix-1: Patient Data Collection Form [file 12913_2023_10287_MOESM1_ESM.docx]

**Appendix-1**

**Patient Data Collection Form**

RN NO.                   DATE            

Case Type: ICU ☐

GENERAL WARDS ☐

Severity: Mild ☐

Moderate ☐

Severe ☐

Asymptomatic: Yes ☐

No ☐

Ward Name: Medical ☐

Surgical ☐

O&G ☐

Orthopaedic ☐

Psychiatric ☐

Others ☐ (Please state): _________

**SECTION ONE: SOCIO DEMOGRAPHIC INFORMATION**

|  |  |  |  |  |  |  |  |  |  |  |  |
| --- | --- | --- | --- | --- | --- | --- | --- | --- | --- | --- | --- |

1. Patient ID

2. Sex: Male ☐ Female ☐

3. Age: ________________ or Date of Birth: Day Month Year

4. Nationality:

Kuwaiti ☐ Non-Kuwaiti ☐

5. Marital status

Single ☐ Married ☐ Divorced ☐

Others ☐ (Please state): _________________

6. Level of Education

Primary school ☐ Secondary school ☐ Diploma ☐ Degree ☐

Others ☐ (Please state): _________________

7. Residence

Capital City ☐ Farwaniya ☐ Hawally ☐

Jahra ☐ Ahmadi ☐ Mubarak Alkabeer ☐

**SECTION TWO: MEDICAL BACKGROUND**

8. Date of admission to Hospital: ______/____/_______

9. Date of discharge from Hospital: _____/___/_________

10. Symptoms on Admission:

| **Symptoms** | **Yes** | **No** |
| --- | --- | --- |
| Fever |  |  |
| Cough |  |  |
| Shortness of Breath/Breathing Difficulty |  |  |
| Sore Throat |  |  |
| Muscle Pain |  |  |
| Running Nose |  |  |
| Fatigue |  |  |
| Abdominal Pain |  |  |
| Headache |  |  |
| Diarrhea |  |  |
| Others: (Please State) |  |  |
| Others: (Please State) |  |  |
| Others: (Please State) |  |  |

11. Date of Admission to ICU: ______/_______/________

12. Date of Discharge from ICU:

13.Number of Days on Mechanical Ventilator:

14. Discharge Status: a) Recovered ☐

b) Transfer to other hospital ☐

c) Died ☐

d) Others: Please Specify_________

**15. Diagnosis**

| No | Types of Diagnosis | Name of Diagnosis | ICD-10 codes (WHO) |
| --- | --- | --- | --- |
| 1 | Primary Diagnosis |  |  |
| 2 | Secondary Diagnosis 1 |  |  |
| 3 | Secondary Diagnosis 2 |  |  |
| 4 | Secondary Diagnosis 3 |  |  |
| 5 | Secondary Diagnosis 4 |  |  |
| 6 | Secondary Diagnosis 5 |  |  |
| 7 | Secondary Diagnosis 6 |  |  |
| 8 | Secondary Diagnosis 7 |  |  |
| 9 | Secondary Diagnosis 8 |  |  |
| 10 | Secondary Diagnosis 9 |  |  |
| 11 | Secondary Diagnosis 10 |  |  |

**16. Procedures:**

| No | Procedure name | Procedure Codes (ICD-9-CM) |
| --- | --- | --- |
| 1 |  |  |
| 2 |  |  |
| 3 |  |  |
| 4 |  |  |
| 5 |  |  |
| 6 |  |  |
| 7 |  |  |
| 8 |  |  |
| 9 |  |  |
| 10 |  |  |

**17. Laboratory Investigations**

| **Type of laboratory investigations** | | Count [*††††*] |
| --- | --- | --- |
| **1** | PCR for COVID-19 |  |
| **2.** | Elisa/RapidTest for COVID-19 |  |
| **3.** | General |  |
|  | Full blood count |  |
|  | Full blood picture |  |
|  | Haemoglobin |  |
|  | MCV or MCHC or MCH |  |
|  | PCV [haematocrit value] |  |
|  | Platelet count |  |
|  | Sedimentation rate [ESR] |  |
|  | Total White [TW] |  |
|  | Total white and differential count [TWBC] |  |
| **4** | Blood |  |
|  | Albumin, blood |  |
|  | Glucose |  |
|  | Liver function tests |  |
|  | Renal profile |  |
|  | Thyroid Profile [Free T4, TSH] |  |
| **5** | **Urine** |  |
|  | FEME |  |
|  | Morphine/Cannabis |  |
| **6** | Cerebrospinal fluid |  |
|  | CSF Biochemistry |  |
|  | Cell count |  |
| **7** | **Pathological examinations** |  |
|  | Cytological examination inclusive of collection specimen |  |
|  | Fine needle aspiration biopsy |  |
|  |  |  |
| 8 | **Other laboratory investigations not listed above**  *[specify below ]* |  |
|  |  |  |
|  |  |  |
|  |  |  |
|  |  |  |
|  |  |  |
|  |  |  |

**18. Radiology Investigation**

| **Type of imaging** | | Tally count [*††††*] | No.  of imaging |
| --- | --- | --- | --- |
| 1 | Plain X Ray |  |  |
| 2 | Ultrasound |  |  |
| 3 | CT scan without contrast |  |  |
| 4 | CT scan with contrast |  |  |
| 5 | MRI scan with contrast |  |  |
| 6 | MRI scan without contrast |  |  |
| 7 | Other diagnostic imaging not listed above  *[specify below ]* |  |  |
|  |  |  |  |
| TOTAL NO OF IMAGING | | | |

**19. Other Investigations**

| No | | Type of laboratory investigations | | Tally count [*††††*] | | Total no of investigation | |
| --- | --- | --- | --- | --- | --- | --- | --- |
| 1 |  | EEG |  | |  | |  |
| 2 |  | Electrocardiogram [ECG] |  | |  | |  |
| 3 |  | Psychological Tests |  | |  | |  |
| 4 |  | Others [specify] |  | |  | |  |
|  |  |  |  | |  | |  |
|  |  |  |  | |  | |  |
|  |  |  |  | |  | |  |
|  |  |  |  | |  | |  |
|  |  |  |  | |  | |  |
|  |  |  |  | |  | |  |

**20. Treatment Oral**:

| **Name of Drugs** | | Quantity  [No. of tablet] | Frequency/day | Duration [days] | Total given |
| --- | --- | --- | --- | --- | --- |
| 1. |  |  |  |  |  |
| 2. |  |  |  |  |  |
| 3 |  |  |  |  |  |
| 4 |  |  |  |  |  |
| 5 |  |  |  |  |  |
| 6 |  |  |  |  |  |
| 7 |  |  |  |  |  |
| 8 |  |  |  |  |  |
| 9 |  |  |  |  |  |
| 10 |  |  |  |  |  |

**21. Treatment Parenteral**:

| **Name of drugs** | | Quantity  [No. of tablet] | Frequency/day | Duration [days] | Total given |
| --- | --- | --- | --- | --- | --- |
| 1 |  |  |  |  |  |
| 2 |  |  |  |  |  |
| 3 |  |  |  |  |  |
| 4 |  |  |  |  |  |
| 5 |  |  |  |  |  |
| 6 |  |  |  |  |  |
| 7 |  |  |  |  |  |
| 8 |  |  |  |  |  |
| 9 |  |  |  |  |  |

| **REMARKS** |
| --- |
|  |
|  |
|  |
|  |
|  |
|  |
|  |
|  |
|  |
|  |
|  |
