# Supplementary material for: Cost of in-patient management of COVID-19 patients in a general hospital in Kuwait
Source: BMC Health Serv Res. 2023 Nov 28;23:1314. doi: 10.1186/s12913-023-10287-z (PMC10685622; doi:10.1186/s12913-023-10287-z)
Supplement: Supplementary file 2 — Appendix-2: Costing Data Collection Tool [file 12913_2023_10287_MOESM2_ESM.docx]

**Appendix-2: Costing Data Collection Tool**

| **Cost Centre** | **No of Staff** | **No of Nurses** | **Inpatient Days** | **Number of Discharges** | **Nos. of Visits** | **ALOS** | **Operating Cost (Exc. Salary)** | **Annual Staff Salary** | **Equipment/furnitures/Vehicles purchased last 5 Years** | **Floor Area (m^2^)** | **Annual Total Cost (KD)** |
| --- | --- | --- | --- | --- | --- | --- | --- | --- | --- | --- | --- |
|  | **2021** | **2021** | **2021** | **2021** | **2021** | **2021** | **2021** | **2021** | **2017-2021** | **2021** | **2021** |
| **A. Overhead Cost Centre** |  |  |  |  |  |  |  |  |  |  |  |
| **1. Administration** | **x** |  |  |  |  |  |  | **x** | **x** | **x** | **x** |
| **2. Nursing Admin** | **x** |  |  |  |  |  |  | **x** | **x** | **x** | **x** |
| **3. Medical Welfare** | **x** |  |  |  |  |  |  | **x** | **x** | **x** | **x** |
| **4. Maintenance** | **x** |  |  |  |  |  |  | **x** | **x** | **x** | **x** |
| **5. Utility** |  |  |  |  |  |  |  |  |  |  | **x** |
| **6. Cleaning Services** | **x** |  |  |  |  |  |  | **x** | **x** | **x** | **x** |
| **7. Security** | **x** |  |  |  |  |  |  | **x** | **x** | **x** | **x** |
| **8. General Store &**  **Consumable** | **x** |  |  |  |  |  |  | **x** | **x** | **x** | **x** |
| **9. IT Centre** | **x** |  |  |  |  |  |  | **x** | **x** | **x** | **x** |
| **10. Library** | **x** |  |  |  |  |  |  | **x** | **x** | **x** | **x** |
| **11. Tax and insurance** |  |  |  |  |  |  |  |  |  |  | **x** |
| **12. Rent** |  |  |  |  |  |  |  |  |  |  | **x** |
| **13. CSSD** | **x** |  |  |  |  |  |  | **x** | **x** | **x** | **x** |
| **14. Dietetic** | **x** |  |  |  |  |  |  | **x** | **x** | **x** | **x** |
| **15. Medical record** | **x** |  |  |  |  |  |  | **x** | **x** | **x** | **x** |
| **16. Laundry & Linen** | **x** |  |  |  |  |  |  | **x** | **x** | **x** | **x** |
| **17. Telephone and Fax** |  |  |  |  |  |  |  |  |  |  | **x** |
| **18. Others** | **x** |  |  |  |  |  |  | **x** | **x** | **x** | **x** |
| **Intermediate Cost Centers** | **No of Staff** | **No of Nurses** | **Inpatient Days** | **Number of Discharges** | **Nos. of Visits** | **ALOS** | **Operating Cost (Exc. Salary)** | **Annual Staff Salary** | **Equipment/furnitures/Vehicles purchased last 5 Years** | **Floor Area (m^2^)** | **Annual Total Cost (KD)** |
| **19. Pharmacy & Drug** | **x** |  |  |  |  |  |  | **x** | **x** | **x** | **x** |
| **20. Radiology** | **x** |  |  |  |  |  |  | **x** | **x** | **x** | **x** |
| **21. Laboratory** | **x** |  |  |  |  |  |  | **x** | **x** | **x** | **x** |
| **22. Physiotherapy** | **x** |  |  |  |  |  |  | **x** | **x** | **x** | **x** |
| **23. ICU** | **x** | **x** | **x** | **x** |  | **x** | **x** | **x** | **x** | **x** | **x** |
| **24. NICU/PICU** | **x** | **x** | **x** | **x** |  | **x** | **x** | **x** | **x** | **x** | **x** |
| **25. CCU & CRW** | **x** | **x** | **x** | **x** |  | **x** | **x** | **x** | **x** | **x** | **x** |
| **26. HDW-Medical** | **x** | **x** | **x** | **x** |  | **x** | **x** | **x** | **x** | **x** | **x** |
| **27. HDW-General** | **x** | **x** | **x** | **x** |  | **x** | **x** | **x** | **x** | **x** | **x** |
| **28. Operation Theatre*** | **x** | **x** |  |  |  |  |  | **x** | **x** | **x** | **x** |
| **29. Operation Theatre**  **(O&G)** | **x** | **x** |  |  |  |  |  | **x** | **x** | **x** | **x** |
| **30. Others** | **x** | **x** | **x** | **x** |  | **x** | **x** | **x** | **x** | **x** | **x** |
|  |  |  |  |  |  |  |  |  |  |  |  |
|  |  |  |  |  |  |  |  |  |  |  |  |
|  |  |  |  |  |  |  |  |  |  |  |  |
|  |  |  |  |  |  |  |  |  |  |  |  |
|  |  |  |  |  |  |  |  |  |  |  |  |
| **Final Cost Centers** | **No of Staff** | **No of Nurses** | **Inpatient Days** | **Number of Discharges** | **Nos. of Visits** | **ALOS** | **Operating Cost (Exc. Salary)** | **Annual Staff Salary** | **Equipment/furnitures/Vehicles purchased last 5 Years** | **Floor Area (m^2^)** | **Annual Total Cost (KD)** |
| **Inpatient Department** |  |  |  |  |  |  |  |  |  |  |  |
| **31. Medicine** | **x** | **x** | **x** | **x** |  | **x** | **x** | **x** | **x** | **x** | **x** |
| **32. Surgical** | **x** | **x** | **x** | **x** |  | **x** | **x** | **x** | **x** | **x** | **x** |
| **33. Pediatric and Nursery** | **x** | **x** | **x** | **x** |  | **x** | **x** | **x** | **x** | **x** | **x** |
| **34. O & G** | **x** | **x** | **x** | **x** |  | **x** | **x** | **x** | **x** | **x** | **x** |
| **35. Orthopedic** | **x** | **x** | **x** | **x** |  | **x** | **x** | **x** | **x** | **x** | **x** |
| **36. Psychiatric** | **x** | **x** | **x** | **x** |  | **x** | **x** | **x** | **x** | **x** | **x** |
| **37. ENT** | **x** | **x** | **x** | **x** |  | **x** | **x** | **x** | **x** | **x** | **x** |
| **38. Ophthalmology** | **x** | **x** | **x** | **x** |  | **x** | **x** | **x** | **x** | **x** | **x** |
| **39. Neurology & Neuro**  **Surgical** | **x** | **x** | **x** | **x** |  | **x** | **x** | **x** | **x** | **x** | **x** |
| **40. Dermatology** | **x** | **x** | **x** | **x** |  | **x** | **x** | **x** | **x** | **x** | **x** |
| **41. Urology** | **x** | **x** | **x** | **x** |  | **x** | **x** | **x** | **x** | **x** | **x** |
| **42. Plastic Surgery** | **x** | **x** | **x** | **x** |  | **x** | **x** | **x** | **x** | **x** | **x** |
| **43. Radiotherapy &**  **Oncology** | **x** | **x** | **x** | **x** |  | **x** | **x** | **x** | **x** | **x** | **x** |
| **44. Cardiothoracic** | **x** | **x** | **x** | **x** |  | **x** | **x** | **x** | **x** | **x** | **x** |
| **46. Respiratory** | **x** | **x** | **x** | **x** |  | **x** | **x** | **x** | **x** | **x** | **x** |
| **47. Rehabilitation** | **x** | **x** | **x** | **x** |  | **x** | **x** | **x** | **x** | **x** | **x** |
| **48. Others** | **x** | **x** | **x** | **x** |  | **x** | **x** | **x** | **x** | **x** | **x** |
| **Outpatient Department** | **No of Staff** | **No of Nurses** | **Inpatient Days** | **Number of Discharges** | **Nos. of Visits** | **ALOS** | **Operating Cost (Exc. Salary)** | **Annual Staff Salary** | **Equipment/furnitures/Vehicles purchased last 5 Years** | **Floor Area (m^2^)** | **Annual Total Cost (KD)** |
| **49. A& E** | **x** | **x** |  |  | **x** |  |  | **x** | **x** | **x** | **x** |
| **50. Medicine Clinic** | **x** | **x** |  |  | **x** |  |  | **x** | **x** | **x** | **x** |
| **51. Surgical Clinic** | **x** | **x** |  |  | **x** |  |  | **x** | **x** | **x** | **x** |
| **52. Pediatric Clinic** | **x** | **x** |  |  | **x** |  |  | **x** | **x** | **x** | **x** |
| **53. O & G Clinic** | **x** | **x** |  |  | **x** |  |  | **x** | **x** | **x** | **x** |
| **54. Orthopedic Clinic** | **x** | **x** |  |  | **x** |  |  | **x** | **x** | **x** | **x** |
| **55. Psychiatric Clinic** | **x** | **x** |  |  | **x** |  |  | **x** | **x** | **x** | **x** |
| **56. ENT Clinic** | **x** | **x** |  |  | **x** |  |  | **x** | **x** | **x** | **x** |
| **57. Dermatology Clinic** | **x** | **x** |  |  | **x** |  |  | **x** | **x** | **x** | **x** |
| **58. Ophthalmology Clinic** | **x** | **x** |  |  | **x** |  |  | **x** | **x** | **x** | **x** |
| **59. Maxillo-facial Clinic** | **x** | **x** |  |  | **x** |  |  | **x** | **x** | **x** | **x** |
| **60. Neurosurgery Clinic** | **x** | **x** |  |  | **x** |  |  | **x** | **x** | **x** | **x** |
| **62. Hemodialysis** | **x** | **x** |  |  | **x** |  |  | **x** | **x** | **x** | **x** |
| **63. Others** | **x** | **x** |  |  | **x** |  |  | **x** | **x** | **x** | **x** |
| **Total** | **x** | **x** | **x** | **x** | **x** | **x** | **x** | **x** | **x** | **x** | **x** |
| **Notes: CSSD (Central Sterile Supply Department), ICU (Intensive Care Unit), NICU (Neonatal Intensive Care Unit), PICU (Pediatric Intensive Care Unit), CCU (Coronary Care Unit), CRW (Coronary Recovery Ward), HDW (High Dependency Ward), O&G (Obstetric & Genecology), ENT (Ear, Nose & Throat), A&E (Accident & Emergency), ALOS (Average Length of Stay)** | | | | | | | | | | | |
